# Impact of Plant Growth-Promoting Rhizobacteria Inoculation and Grafting on Tolerance of Tomato to Combined Water and Nutrient Stress Assessed via Metabolomics Analysis

**DOI:** 10.3389/fpls.2021.670236

**Published:** 2021-06-04

**Authors:** Panagiotis Kalozoumis, Dimitrios Savvas, Konstantinos Aliferis, Georgia Ntatsi, George Marakis, Evridiki Simou, Anastasia Tampakaki, Ioannis Karapanos

**Affiliations:** ^1^Department of Crop Science, Laboratory of Vegetable Production, Agricultural University of Athens, Athens, Greece; ^2^Department of Crop Science, Laboratory of Pesticide Science, Agricultural University of Athens, Athens, Greece; ^3^Department of Plant Science, McGill University, Macdonald Campus, Sainte-Anne-de-Bellevue, QC, Canada; ^4^Department of Crop Science, Laboratory of General and Agricultural Microbiology, Agricultural University of Athens, Athens, Greece

**Keywords:** biostimulant, hydroponics, grafting, metabolomics, M82, PGPR, tomato, water stress

## Abstract

In the current study, inoculation with plant growth-promoting rhizobacteria (PGPR) and grafting were tested as possible cultural practices that may enhance resilience of tomato to stress induced by combined water and nutrient shortage. The roots of tomato grown on perlite were either inoculated or not with PGPR, applying four different treatments. These were PGPR-T1, a mix of two *Enterobacter* sp. strains (C1.2 and C1.5); PGPR-T2, *Paenibacillus* sp. strain DN1.2; PGPR-T3, *Enterobacter mori* strain C3.1; and PGPR-T4, *Lelliottia* sp. strain D2.4. PGPR-treated plants were either self-grafted or grafted onto *Solanum lycopersicum* cv. M82 and received either full or 50% of their standard water, nitrogen, and phosphorus needs. The vegetative biomass of plants subjected to PGPR-T1 was not reduced when plants were cultivated under combined stress, while it was reduced by stress to the rest of the PGPR treatments. However, PGPR-T3 increased considerably plant biomass of non-stressed tomato plants than did all other treatments. PGPR application had no impact on fruit biomass, while grafting onto ’M82’ increased fruit production than did self-grafting. Metabolomics analysis in tomato leaves revealed that combined stress affects several metabolites, most of them already described as stress-related, including trehalose, myo-inositol, and monopalmitin. PGPR inoculati*o*n with *E. mori* strain C3.1 affected metabolites, which are important for plant/microbe symbiosis (myo-inositol and monopalmitin). The rootstock M82 did not affect many metabolites in plant leaves, but it clearly decreased the levels of malate and D-fructose and imposed an accumulation of oleic acid. In conclusion, PGPR are capable of increasing tomato tolerance to combined stress. However, further research is required to evaluate more strains and refine protocols for their application. Metabolites that were discovered as biomarkers could be used to accelerate the screening process for traits such as stress tolerance to abiotic and/or abiotic stresses. Finally, ‘M82’ is a suitable rootstock for tomato, as it is capable of increasing fruit biomass production.

## Introduction

The Mediterranean region, where tomato is widely cultivated, is expected to be strongly affected by the climate change in the following years (Giorgi, 2006; Giorgi and Lionello, 2008). One of the consequences of climate change in the Mediterranean area is the decrease of the average yearly precipitation, which leads to water scarcity, a situation already familiar to many Mediterranean countries (Saadi et al., 2015). Tomato is a water-demanding crop (Ngouajio et al., 2007), and, consequently, water deficit can result in severe yield decreases compared with cultivation under fully irrigated conditions (Kuşçu et al., 2014). Additionally, irrigation along with other parameters, including fertilization, affects the nutritional composition of tomato fruits (Smith and Hui, 2004). To cope with this situation, it is crucial to develop new cropping approaches contributing to reduced water consumption. Deficit irrigation has been postulated as an alternative irrigation strategy that might save water without or with minimal consequences on crop yield, but conclusions as to whether the concomitant yield losses are affordable do not converge (Giuliani et al., 2016).

In addition to water deficit, excessive fertilizer application in intensive agriculture and horticulture also raises environmental concerns, especially in developed countries, but also in developing countries where an increased phosphorus demand is recorded (Desmidt et al., 2015). The initial fears about the availability of P resources are currently revised, and many studies suggest that natural P reserves will not be depleted in the next 100 years (Günther, 1997; Cisse and Mrabet, 2004; Van Vuuren et al., 2010). However, if the plant-available P in the Earth’s crust is depleted, access to discrete global reserves is politically sensitive, energy demanding, and economically challenging (Godfray et al., 2010; Neset and Cordell, 2012; Desmidt et al., 2015). Similarly, the use of N fertilizers is associated with increased greenhouse gas emissions, thereby contributing to climate change, firstly because their production through industrial N_2_ fixation entails extensive consumption of fossil fuels (Chen et al., 2018) and secondly because part of the soil NO_3_–N is converted into N_2_O in the soil (Smith and Zimmerman, 1981). Thus, crop fertilization strategies associated with reduced application of N and P fertilizers are strongly supported by current European policies (EU Directive 889/2008).

Plant growth-promoting rhizobacteria (PGPR) application is considered an effective strategy to stimulate plant growth, while modulating abiotic stress responses via a multitude of mechanisms (Patakioutas et al., 2015; Ruzzi and Aroca, 2015; Backer et al., 2018; Rouphael and Colla, 2020). Plant growth promotion by PGPR is the outcome of different mechanisms, such as biological nitrogen fixation, ethylene levels reduction, siderophore production, phytohormone production, induction of pathogen resistance, nutrient solubilization, mycorrhizal functioning, and decreasing pollutant toxicity (Glick et al., 1999; Rouphael and Colla, 2020). Nutrient solubilization can lead to production of biofertilizers, particularly for solubilizing phosphorus (De Pascale et al., 2018) where resources are limited (Bhattacharyya and Jha, 2012). Induced systemic resistance can also provide an effective pathogen protection even in organic agriculture (Ramamoorthy et al., 2001). Thus, inoculation of plant roots with a mix of different growth-promoting bacteria strains is considered a very promising technique to protect plants from diseases and simultaneously enhance plant growth (Murphy et al., 2003).

Many different PGPR strains have been already tested in tomato crops under different soil or soilless conditions (De Brito et al., 1995; Yan et al., 2003). Colonization of tomato roots with PGPR is performed by inoculating the seeds or by applying the bacteria suspension thoroughly into the soil/soilless medium with no need of sterilization (Yan et al., 2003). Many studies with tomato showed that PGPR inoculation increases yield (Gagné et al., 1993), lycopene, antioxidants, and potassium in tomato fruits (Ordookhani et al., 2010). Furthermore, PGPR increased P levels in tomato shoots (Hariprasad and Niranjana, 2009) and contributed to more efficient control of nematode and pathogen infections (Seleim et al., 2011; Shanmugam and Kanoujia, 2011; Almaghrabi et al., 2013).

Grafting is an alternative technique that can confer enhanced tolerance to tomato under both biotic and abiotic stress conditions (Lee and Oda, 2003; Rouphael et al., 2017). Amelioration of abiotic stress imposed by shortage of nutrients or water has been reported by several scientists, including Rouphael et al. (2008a, b), Savvas et al. (2010), Schwarz et al. (2010), Colla et al. (2011), and Rouphael et al. (2017). Some rootstocks proved to be capable of mitigating nutrient shortage stress by increasing the nutrient-to-water uptake ratio, thereby resulting in enhanced nutrient translocation to the shoot (Savvas et al., 2017). Nevertheless, for some other nutrients, increased nutrient-to-water uptake ratios occur merely because of their enhanced deposition to the rootstock.

Stress due to shortage of water also restricts plant growth and crop yield due to impairment of the plant metabolism. Drought stress imposes generation of reactive oxygen species during several metabolic processes (Sánchez-Rodríguez et al., 2011). Other important metabolites affected by water deficit are sugar and sugar derivatives (Dumville and Fry, 2002; Semel et al., 2007) and amino acids (Semel et al., 2007), where changes in proline and glutamate content have been recognized as responses to drought stress (Zhu, 2000; Bartels and Sunkar, 2005; Semel et al., 2007). Fatty and organic acids generally increase as a response to drought stress, especially those occurring as intermediates of the tricarboxylic acid (TCA) cycle.

Scarcity of good-quality irrigation water and high demand of energy in fertilizer production, combined with reduced availability of plant nutrient resources, impose increasing pressure to horticulture to adopt water- and nutrient-saving cultural practices with no or minimal yield losses. To address this challenge, inoculation with PGPR and grafting onto suitable rootstocks were tested in the current study as possible cultural practices that may enhance resilience of greenhouse tomato to stress induced by combined water and nutrient (N and P) shortage. To contribute to the discovery of potential biomarkers that might accelerate the progress of screening new effective biostimulants and rootstocks, the impact on plant metabolism was studied by applying metabolomics analysis, in addition to growth and yield responses.

## Materials and Methods

### Plant Material, Growth Conditions, and Treatments

The experiment was conducted during the winter of 2017 in a glasshouse at the Agricultural University of Athens (N 37°59′10″, E 23°42′29″, altitude 24 m). T omato (*Solanum lycopersicum*) cv. Belladonna F1 was grown in an open hydroponic system using perlite as growing medium, placed in pots. Plants were either self-grafted (Belladonna/Belladonna) or grafted onto tomato *S. lycopersicum* Mill. cv. M82. The tomato cultivar M82, which has been previously studied under water deficit conditions (Rigano et al., 2016; Illouz-Eliaz et al., 2020), was selected because it has been used for backcrossing with *Solanum pennelli* aiming to produce water-stress-tolerant tomato rootstocks (Lippman et al., 2007; Ofner et al., 2016).

Tomato seedlings were transplanted into the greenhouse on November 23, 2018, at the stage of five true leaves. Before being transplanted, the growing medium around the roots of the seedlings was removed, and the roots were placed for 1 min into a PGPR suspension containing 10^9^ CFU ml^–1^, which was diluted 10-fold before use. The inoculation of tomato seedlings was repeated 5 days later by drenching the growing medium with the same PGPR suspension using a 5-ml pipette. Five PGPR strains were used to establish the following four PGPR treatments: PGPR-T1, a mix of two *Enterobacter* sp. strains (C1.2 and C1.5); PGPR-T2, *Paenibacillus* sp. strain DN1.2; PGPR-T3, *Enterobacter mori* strain C3.1; and PGPR-T4, *Lelliottia* sp. strain D2.4. These PGPR treatments were complemented with a control treatment in which no PGPR was applied to the tomato roots. The bacterial strains used in the present study belong to bacterial genera or species known to exhibit PGP traits according to the literature (Jha et al., 2011; Amaresan et al., 2020; Orozco-Mosqueda and Gustavo, 2020). Moreover, the strains showed tolerance in the presence of 8% (w/v) NaCl (Tampakaki et al., unpublished results), and for this reason, they were considered suitable for testing under drought conditions.

Plant density was 2.4 plants m^–2^ at the beginning of the experiment. The experiment was set up as a three-factorial (2 rootstocks × 5 PGPR × 2 stress treatments), completely randomized block design with three replicates per treatment and three plants per replicate. A bumblebee hive was placed into the glasshouse on December 19, 2017, to secure optimal pollination. On January 8, 2018, i.e., 6 weeks after transplanting, one plant per replicate was sampled and used to estimate the root and the aboveground biomass production. After removal of these plants, the plant density decreased to 1.6 plants m^–2^. Harvest commenced on February 9, 2018; and the experiment was terminated on March 30, 2018. Air temperature was always maintained to levels above 13°C during the night and above 18°C during the day.

Combined stress was applied by reducing water and nutrient (N and P) supply to 50% of the standard supply in the non-stress treatment. The daily amount of supplied nutrient solution (NS) differed among stressed and non-stressed treatments. Non-stressed treatments were fertigated using a solar meter to control irrigation frequency aiming to obtain a drainage fraction of 20% of the total NS amount supplied to plants. The irrigation frequency in the stressed plants was similar with that applied to the non-stress plants, but the amount of NS provided at each irrigation event was half as much as that provided to the non-stressed plants. Additionally, concentrations of nitrogen and phosphorus in the NS supplied to the stressed plants were half as high as in the standard NS for tomato (Savvas et al., 2013) provided to the non-stressed plants. Nutrient concentrations in the NS are provided in Supplementary Table 1.

### Biomass and Total Yield Determination

Aboveground biomass samples were obtained 6 weeks after transplanting and at the end of the cultivation, by harvesting the entire shoot. Root fresh biomass was also measured at the first sampling. After the tomato shoot was harvested, fresh weight was recorded, and samples were oven-dried at 65°C for at least 72 h, until constant weight was achieved, and used to determine their dry weight and mineral composition. The impact of the experimental treatments on fruit yield was assessed by manually harvesting three times a week all commercially ripe fruits commencing on February 9, 2018, and terminating on March 30, 2018. Fruits were also classified into four classes (Extra Class, Class I, Class II, and non-marketable) as described in the Eu Regulation (543/2011).

### Mineral Analysis

The dried samples of tomato leaves were powdered using a blade mill and passed through a 40-mesh sieve. Plant tissue samples were used for chemical analysis to determine total N, P, and K concentrations. Total N was determined applying the Kjeldahl method. To estimate P and K, leaf samples were milled and dry ashed at 550°C for 5 h, and the ash was dissolved in 1 M of HCl. Phosphorus was measured photometrically as phosphomolybdate blue complex at 880 nm using a 96-position microplate spectrophotometer (Anthos Zenyth 200; Biochrom, United States). Potassium was determined using a flame photometer (Sherwood Model 410, Cambridge, United Kingdom).

### Gas Chromatography/Electron Impact/Mass Spectrometry Metabolomics Analysis of Tomato Leaves

#### Chemicals and Reagents

For the derivatization of samples for gas chromatography/electron impact/mass spectrometry (GC/EI/MS) analysis, the reagents ribitol, methoxylamine hydrochloride, *N*-methyl-*N*-(trimethyl-silyl)trifluoroacetamide (MSTFA), and pyridine were used (Sigma-Aldrich Ltd., Steinheim, Germany). The organic solvents methanol and ethyl acetate (Carlo Erba Reagents, Val de Reuil, France) were used for the extraction of leaf tissues.

#### Plant Material and Sample Preparation

Entire young, fully expanded, and healthy tomato leaves were collected and placed directly in liquid nitrogen for quenching and then were stored at −80°C until further processing. Samples were collected from plants 55 days following transplantation. Metabolomics was applied to non-stressed and stressed plants and to self-grafted and grafted M82 plants. Furthermore, based on the results of early plant biomass measurements, only samples of plants from the PGPR-T3 and non-inoculated plants were subjected to metabolomics.

#### Metabolite Extraction for Gas Chromatography/Electron Impact/Mass Spectrometry Analysis

Tomato leaves were pulverized using pestle and mortar in liquid nitrogen, and the pulverized tissues were placed in Eppendorf tubes (2 ml) and stored in −80°C. For metabolomics, 50 mg was transferred into Eppendorf tubes (2 ml); and 1 ml of a solution of methanol:ethyl acetate (50:50 v/v) and 20 μl of ribitol (0.2 mg ml^–1^ methanol), serving as internal standard, were added. A previously described protocol was used (Kostopoulou et al., 2020), with minor modifications. Samples that were obtained by pooling portions of the biological replications of each treatment served as the quality-control samples. The resulting suspensions were sonicated for 20 min in an ultrasonic bath (Branson 1210, Danbury, United States) and then agitated using a horizontal rotary shaker (GFL 3006, Gesellschaft für Labortechnik mbH, Burfwedel, Germany) at 150 rpm for 2 h. The suspensions were filtered using PSTFA filters (0.2-μm pore diameter, Macherey-Nagel, Duren, Germany). Derivatization of samples for GC/EI/MS analysis was performed in a two-step process using methoxylamine hydrochloride in pyridine (20 mg ml^–1^) for methoxymation and MSTFA for silylation. The derivatized samples were finally transferred to microinserters (180 μl, Macherey-Nagel).

#### Gas Chromatography–Mass Spectrometry Analyses

The derivatized samples were analyzed by GC/EI/MS (GC/EI/MS Agilent 6890n, Agilent Technologies Inc.) equipped with an inert mass-selective detector 5973 (MSD) and a 7683 autosampler. The electron ionization was set to 70 eV, and full scan mass spectra were acquired at 50–800 Da in a rate of 4 scans per second with a 10-min solvent delay. The temperature for the ion source was set to 150°C and for the transfer line to 230°C. An HP-5MS capillary column (30 m, i.d. 0.25 mm, and film thickness 0.25 μm; Agilent Technologies Inc.) was used, and helium (He 6.0) was the carrier gas. Samples were injected in completely randomized order on column, and the injector split ratio was set to 5:1. The initial temperature of the oven was 70°C, stable for 5 min, followed by an increase of 5°C per min to 295°C.

### Statistical Analysis

The experiment was set as a complete randomized block design with three factors (2 × 5 × 2) and three replications per treatment. Data were analyzed by applying three-way analysis of variance (ANOVA) to assess main effects [rootstocks (R), PGPR, and stress conditions (S)], three first-order interactions (R × PGPR, R × S, and PRPR × S), and one second-order interaction (R × PGPR × S). Multiple comparisons of means were performed by applying the Duncan multiple range test (p < 0.05) after performing three-way ANOVA. The data were statistically analyzed using STATISTICA, version 8.0 (StatSoft, Inc., Tulsa, OK, United States).

For the metabolomics analyses, all experimental events were controlled using the software MSD Chemstation (Agilent). The deconvolution of the acquired total ion chromatograms was performed using the software AMDIS v.2.66 [National Institute of Standards and Technology (NIST); Gaithersburg, MD, United States], and the MS database of the NIST ‘08 (NIST; Gaithersburg, MD, United States). The absolute identification for selected metabolites was performed by matching their retention times and mass fragmentation patterns to those of analytical standards, which had been analyzed employing the same method and analyzer, as recommended by the Metabolomics Standards Initiative (MSI) (Sansone et al., 2007). The tentative annotation of features was performed based on the similarities of their fragmentation patterns to those of entries of the NIST library (>90%). Based on the deconvolution of the data, features present also in the experimental blank samples or features that correspond to column artifacts were excluded from analyses. The data pre-processing, including feature alignment and gap filling, was performed using the software MS-Dial v.3.70, applying the recommended settings for GC/EI/MS (Tsugawa et al., 2015). The discovery of trends and biomarkers was on based on the values of scaled and centered orthogonal partial least square (OPLS) regression coefficients (CoeffCS) (*p* < 0.05). OPLS-discriminant analysis (OPLS-DA) was applied, since it provides optimal model interpretability and transparency (Aliferis and Chrysayi-Tokousbalides, 2011), using the software SIMCA-P v.13.0.3 (Umetrics, Sartorius Stedim Data Analytics AB, Umeå, Sweden), as previously described (Chatzigianni et al., 2020; Karamanou and Aliferis, 2020; Kostopoulou et al., 2020). The hierarchical cluster analysis (HCA) of the data was performed by the Ward linkage method.

## Results

### Biomass Production

**FIGURE 1:**
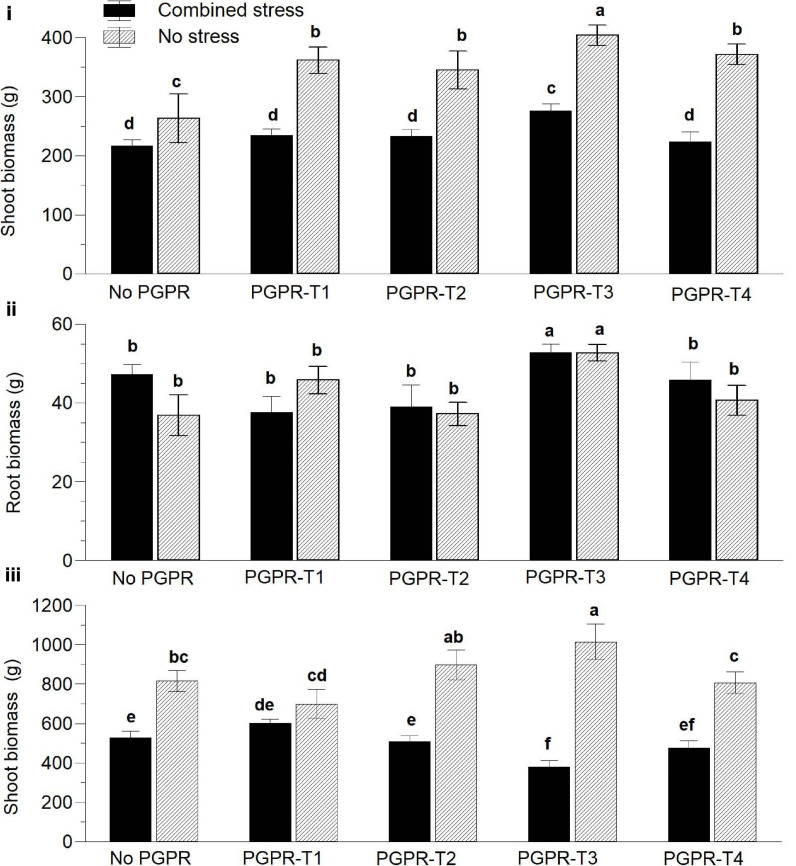
Effects of combined water and nutrient stress (restriction of water, N, and P supply by 50%), and plant growth-promoting rhizobacteria (PGPR) inoculation on aboveground vegetative biomass **(i)** and root biomass **(ii)** 45 days after transplanting and aboveground biomass at the end of the cultivation period **(iii)**. PGPR-T1, a mix of *Enterobacter* sp. strains C1.2 and C1.5; PGPR-T2, *Paenibacillus* sp. strain DN1.2; PGPR-T3, *Enterobacter mori* strain C3.1; and PGPR-T4, *Lelliottia* sp. strain D2.4. Data are means of three replications. Means followed by different letters indicate significant differences according to the Duncan multiple range test (*p* < 0.05).

Six weeks after transplanting, the exposure of tomato to combined nutrient (N and P) and water stress reduced significantly the aboveground fresh biomass compared with non-stressed plants (Figure 1i). Inoculation of tomato with PGPR exerted a positive influence on plant biomass than did non-inoculated plants regardless of the applied PGPR treatment. Among the different PGPR treatments, PGPR-T3 had the highest biomass under both non-stress and combined stress conditions. Plants inoculated with PGPR-T3 exhibited also significantly higher root biomass than did the rest of the PGPR treatments and the non-inoculated plants under both no stress and combined stress conditions (Figure 1ii). Grafting ‘Belladonna’ onto ‘M82’ had no impact on the fresh plant biomass 6 weeks after transplanting (data not presented).

At the end of the experiment, clear differences were again observed between non-stressed plants and plants exposed to combined water and N/P stress, regarding the aboveground fresh biomass. More specifically, non-stressed plants resulted in higher aboveground biomass than did stressed plants (Figure 1iii). The PGPR application also affected the aboveground vegetative biomass, but its impact interacted with the combined water and nutrient stress. Under non-stress conditions, PGPR-T3 resulted in the highest aboveground vegetative biomass, and the difference was significant in comparison not only with the treatment without PGPR application but also with PGPR-T1 and PGPR-T4. However, under combined water and nutrient stress conditions, PGPR-T3 resulted in the lowest aboveground vegetative biomass. Furthermore, the aboveground vegetative biomass was not reduced by combined water and nutrient stress in plants of the PGPR-T1, while it was reduced in all other PGPR treatments.

Plants grafted onto the rootstock M82 exhibited 30% higher aboveground fresh biomass than did self-grafted plants under non-stress conditions. However, under stress conditions imposed by combined nutrient and water shortage, grafting had no impact on the aboveground vegetative biomass (data not presented).

### Tomato Yield

Plants cultivated under combined water and N–P deficit produced fewer fresh fruits by 34.8% than did plants with optimal fertigation (Table 1). The reduction of yield by combined water and nutrient stress originated from decreases in both the mean fruit weight by 15.7% and the number of fruits per plant by 22.7%. Moreover, combined stress restricted also the total weight of fruit graded Extra Class and Class I by 37.9%. Inoculation of tomato roots with PGPR had no impact on total fruit yield and yield components, as well as quality grading. Grafting onto the rootstock M82 did not affect fruit mean weight but significantly increased the number of fruits per plant resulting in 11.6% more total fruit production and a significant increase in Extra Class fruits compared with self-grafting. The effect of grafting on total fruit yield and number of fruits per plant did not interact with the combined water and nutrient stress, while the production of Extra and Class I fruits was enhanced by grafting only under non-stress conditions.

**TABLE 1:** Effects of combined restriction of water, N, and P supply by 50%, PGPR inoculation (PGPR-T1, a mix of *Enterobacter* sp. strains C1.2 and C1.5; PGPR-T2, *Paenibacillus* sp. strain DN1.2; PGPR-T3, *Enterobacter mori* strain C3.1; and PGPR-T4, *Lelliottia* sp. strain D2.4) and grafting (self-grafted plants and grafted plants onto M82) on tomato total fruit production, fruit number, fruit mean weight, and weight of fruits graded Extra Class and Class I.

Treatment	Total fruit production (kg m^–2^)	Number of fruits m^–2^	Mean fruit weight (g)	Extra class and class I (kg m^–2^)
No stress	4.20 a	26.4 a	159.2 a	3.99 a
Stress	2.74 b	20.4 b	134.2 b	2.48 b
No PGPR	3.33	23.3	142.7	3.09
PGPR-T1	3.55	24.4	145.3	3.28
PGPR-T2	3.54	23.7	149.2	3.30
PGPR-T3	3.49	23.5	148.2	3.31
PGPR-T4	3.43	23.2	148.1	1.18
Self-grafted	3.28 b	22.1 b	148.4	3.04 b
Grafted onto M82	3.66 a	25.2 a	145.0	3.43 a
Significant interactions				
No stress × self-grafted				3.65 b
No stress × grafted onto M82				4.34 a
Stress × self-grafted				2.43 c
Stress × grafted onto M82				2.53 c
Statistical significance				
Stress	***	***	***	***
PGPR treatment	ns	ns	ns	ns
Grafting	*	*	ns	*
Stress × PGPR	ns	ns	ns	ns
Stress × grafting	ns	ns	ns	**
PGPR × grafting	ns	ns	ns	ns
Stress × PGPR × grafting	ns	ns	ns	ns

Means (*n* = 3) followed by different letters indicate significant differences according to the Duncan multiple range test (*p* < 0.05), at *p* < 0.05, *p* < 0.01, and *p* < 0.001, denoted by *, **, and ***, respectively; ns, not significant.

*PGPR, plant growth-promoting rhizobacteria.*

### Leaf Nutrient Concentrations

Combined reduction of water, N, and P supply by 50% decreased significantly the total N concentrations in leaves when the roots were not inoculated, or inoculated with PGPR-T1 or PGPR-T3, but had no impact on leaf total-N when inoculated with PGPR-T2 and PGPR-T4 (Table 2). Grafting had no significant impact on the leaf total N concentration. Under non-stress conditions, PGPR inoculation did not affect the leaf total N level, while under combined water and nutrient stress conditions, PGPR-T2 and PGPR-T4 resulted in significantly higher leaf total N levels compared with PGPR-T1.

**TABLE 2:** Effects of combined restriction of water, N, and P supply by 50%, PGPR inoculation (PGPR-T1, a mix of *Enterobacter* sp. strains C1.2 and C1.5; PGPR-T2, *Paenibacillus* sp. strain DN1.2; PGPR-T3, *Enterobacter mori* strain C3.1; and PGPR-T4, *Lelliottia* sp. strain D2.4) and grafting (self-grafted plants and grafted plants onto M82) on total N (g kg^–1^ DM) and P (g kg^–1^ DM) in tomato leaves.

Treatment	N (g kg^–1^ DM)	P (g kg^–1^ DM)
No stress	35.2 a	7.30 a
Stress	32.1 b	2.60 b
No PGPR	32.3 b	5.36
PGPR-T1	31.2 b	4.69
PGPR-T2	37.0 a	4.57
PGPR-T3	33.2 b	5.17
PGPR-T4	34.5 ab	4.99
Self-grafted	34.6	5.33 a
Grafted onto M82	32.7	4.58 b
**Significant interactions**		
No stress × no PGPR	37.0 a	
No stress × PGPR-T1	34.3 a	
No stress × PGPR-T2	36.5 a	
No stress × PGPR-T3	33.6 a	
No stress × PGPR-T4	34.7 a	
Stress × no PGPR	27.0 b	
Stress × PGPR-T1	28.0 b	
Stress × PGPR-T2	37.6 a	
Stress × PGPR-T3	32.9 ab	
Stress × PGPR-T4	34.3 a	
**Statistical significance**		
Stress	**	***
PGPR treatment	*	ns
Grafting	ns	**
Stress × PGPR	*	ns
Stress × grafting	ns	ns
PGPR × grafting	ns	ns
Stress × PGPR × grafting	ns	ns

Means (*n* = 3) followed by different letters indicate significant differences according to the Duncan multiple range test at *p* < 0.05, *p* < 0.01, and *p* < 0.001, denoted by *, **, and ***, respectively; ns, not significant.

*PGPR, plant growth-promoting rhizobacteria.*

The leaf P concentration was significantly reduced by combined water and nutrient stress conditions regardless of grafting and PGPR application (Table 2). Grafting onto M82 reduced slightly but significantly the leaf P concentration from 5.3 to 4.6 mg g^–1^. The leaf K concentration was significantly reduced from 51.4 to 45.2 mg g^–1^ when plants were exposed to combined water and nutrient stress, while grafting and PGPR application had no significant impact on the leaf K (data not presented).

### Metabolomics Analysis

**FIGURE 2:**
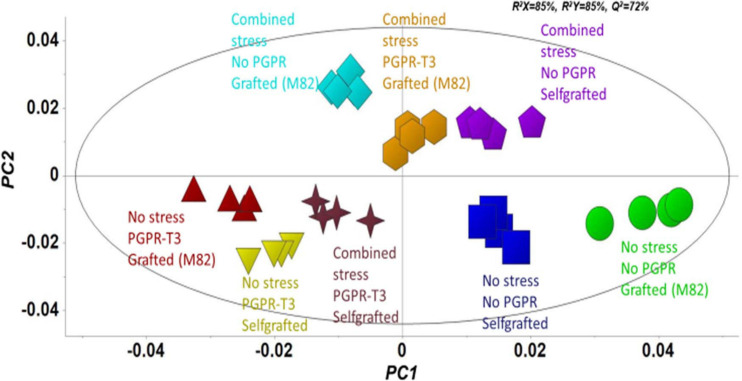
Orthogonal partial least squares-discriminant analysis (OPLS-DA) PC1/PC2 score plot for the gas chromatography/electron impact/mass spectrometry (GC/EI/MS) metabolite profiles of tomato leaves [principal component (PC)]. The ellipse represents the Hotelling T^2^ with 95% confidence interval.

**FIGURE 3:**
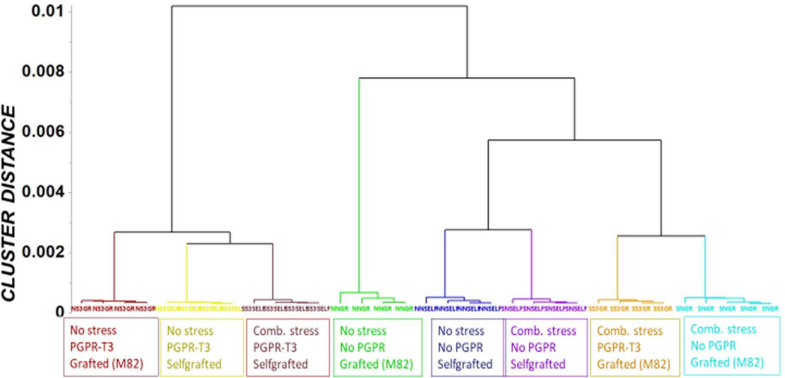
Orthogonal partial least square (OPLS) dendrogram for the recorded gas chromatography/electron impact/mass spectrometry (GC/EI/MS) metabolite profiles of tomato leaves. Cluster distances were calculated using the Ward linkage method.

Fluctuations in tomato leaf metabolome in response to combined water and nutrient stress, PGPR inoculation, and grafting were recorded using bioinformatics software and metabolite species-specific databases. A representative raw GC/EI/MS data set [*S. lycopersicum* L. (Tomato) (PMG-01-21)] can be found at the repository of the Pesticide Metabolomics Group of the Agricultural University of Athens^1^. In total, 167 metabolite features were discovered, and the annotated ones belonging to various chemical groups are presented in Supplementary Data Set 1. The application of OPLS-DA revealed tight groups with no outliers at *p* < 0.05 (Figure 2). The leaf metabolome of tomato plants treated with *E. mori* strain C3.1 (PGPR-T3) was clearly discriminated from that of non-inoculated plants, except for plants grafted onto M82 under combined stress conditions. Non-stressed plants were also clearly discriminated from plants exposed to combined water and nutrient stress, except for stressed plants, which were self-grafted and inoculated with *E. mori* strain C3.1 (Figure 3). The examination of the hierarchical manner of the treatments metabolism generally revealed that PGPR inoculation had the greatest cluster distance from the non-inoculated treatment except of plants inoculated with PGPR-T3, grafted onto ‘M82’ and cultivated under combined stress, which was discriminated from the rest of the PGPR treatments.

**FIGURE 4:**
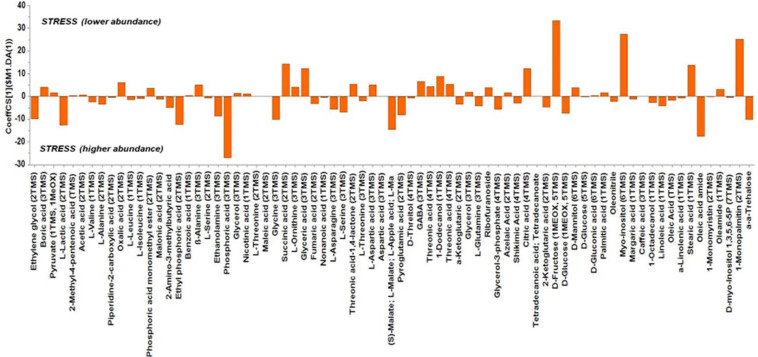
Orthogonal partial least square (OPLS) coefficient plots for the gas chromatography/mass spectrometry (GC/MS) metabolic profiles of tomato leaves performing pairwise comparisons between non-stressed plants and combined stressed plants. The values of scaled and centered OPLS regression coefficients (CoeffCS) are displayed. Influential metabolites for the observed discriminations between the metabolomes of non-stressed and combined stress plants are displayed with jackknifed confidence intervals (*p* < 0.05). Negative values of CoeffCS denote metabolites with higher concentration in combined stressed plants, whereas positive values are those with higher concentration in non-stressed plants.

**FIGURE 5:**
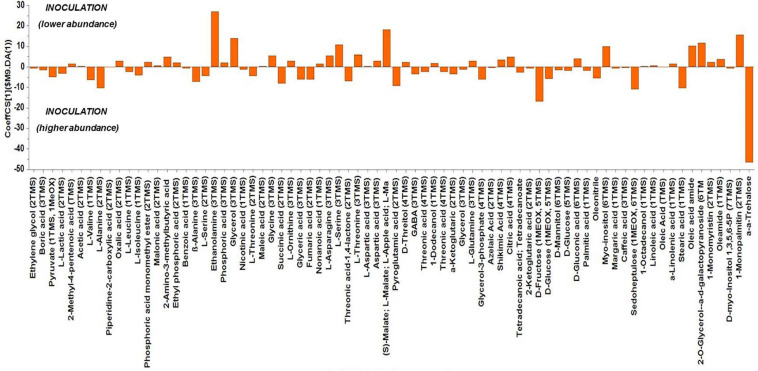
Orthogonal partial least square (OPLS) coefficient plots for the gas chromatography/mass spectrometry (GC/MS) metabolic profiles of tomato leaves performing pairwise comparisons between non-inoculated plants [No plant growth-promoting rhizobacteria (PGPR)] and plants inoculated with PGPR-T3. The values of scaled and centered OPLS regression coefficients (CoeffCS) are displayed. Influential metabolites for the observed discriminations between the metabolomes of non-inoculated plants and plants inoculated with PGPR-T3 are displayed with jackknifed confidence intervals (*p* < 0.05). Negative values of CoeffCS denote metabolites with higher concentration in plants inoculated with PGPR-T3, whereas positive values are those with higher concentration in non-inoculated plants.

**FIGURE 6:**
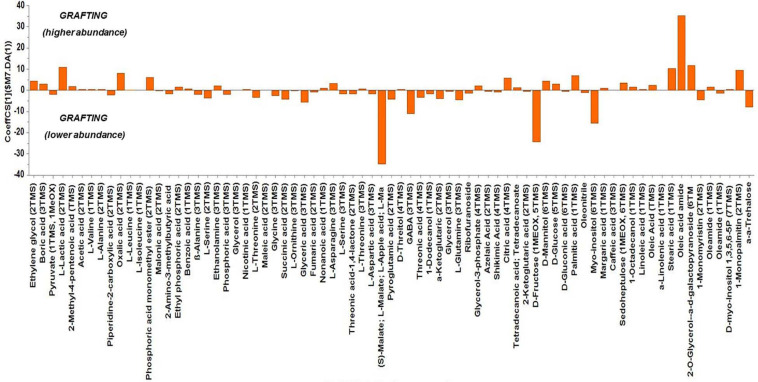
Orthogonal partial least square (OPLS) coefficient plots for the gas chromatography/mass spectrometry (GC/MS) metabolic profiles of tomato leaves performing pairwise comparisons between self-grafted tomato plants (‘Belladonna’ F1) and plants grafted onto the rootstock M82. The values of scaled and centered OPLS regression coefficients (CoeffCS) are displayed. Influential metabolites for the observed discriminations between the metabolomes of self-grafted and grafted plants are displayed with jackknifed confidence intervals (*p* < 0.05). Negative values of CoeffCS denote metabolites with higher concentration in self-grafted plants, whereas positive values are those with higher concentration in plants grafted onto ‘M82’.

Based on the values of the corresponding CoeffCS, the metabolites exhibiting the most marked accumulation, which exceeded a 10-fold increase in plants exposed to combined water, N, and P stress, were ethylene glycol, L-lactic acid, ethyl phosphoric acid, phosphoric acid, glycine, malate, pyroglutaminic acid, oleic acid, and α-α-trehalose, while succinic acid, glyceric acid, citric acid, D-fructose, myo-inositol, stearic acid, and 1-monopalmitin were reduced 10-fold (Figure 4). The PGPR inoculation with PGPR-T3 increased over 10-fold D-fructose and α-α-trehalose levels but decreased ethanolamine, glycerol, malate, myo-inositol, oleic acid, 2-*O*-glycerol-α-D-galactopyranoside, and 1-monopalmitin in tomato leaves (Figure 5). Finally, grafting ‘Belladona’ onto the rootstock ‘M82’ resulted in a more than 10-fold higher relative abundance of stearic acid, oleic acid, 2-*O*-glycerol-α-D-galactopyranoside, and 1-monopalmitin but lower than 10-fold relative abundance of malate, D-fructose, and myo-inositol than in self-grafted ‘Belladonna’ (Figure 6).

## Discussion

### Biomass Production

Plant growth-promoting rhizobacteria can stimulate plant growth through a range of mechanisms, including excretion of substances acting as plant growth regulators or stimulation of their biosynthesis by plants, improved nutrient acquisition, and inhibition of fungal plant pathogens (Raaijmakers et al., 2009; Choudhary et al., 2011). Furthermore, PGPR can induce systemic tolerance to abiotic stress (Yang et al., 2009). In a study with tomato and pepper cultivated under optimal or water stress conditions, inoculation of the roots with PGPR increased biomass production compared with no application of PGPR under both optimal and water stress conditions (Mayak et al., 2004). On the other hand, in an experiment with tomato inoculated with different PGPR strains, Shen et al. (2012) found that PGPR-induced growth promotion occurred only under stress conditions imposed by simulated seawater irrigation. These contradictory results indicate that the impact of PGPR application on cultivated plants is species- and strain-specific, as different bacteria possess different growth stimulation mechanisms, as also suggested by Dey et al. (2004).

Although the use of PGPR on tomato cultivation has been already addressed in several studies, the PGPR strains applied in the current study have never been tested in the past as PGPR. All PGPR treatments applied in the current study enhanced the root and shoot growth of tomato under non-stress conditions during the early vegetative developmental stage. However, *E. mori* strain C3.1 (PGPR-T3) increased more markedly the root and shoot biomass than the other three PGPR treatments, compared with no application of PGPR. Furthermore, both the root and shoot biomass of plants treated with *E. mori* strain C3.1 were not reduced by application of combined nutrient/water stress for 6 weeks, compared with those of plants not treated with PGPR.

Despite the protective role of PGPR-T3 on tomato growth during the early cultivation stage, PGPR-T3 resulted in the lowest shoot biomass under combined nutrient and water stress conditions at crop termination. These results indicate that the protective effect of *E. mori* strain C3.1 was attenuated during the cropping period. Since PGPR was applied only once at crop establishment, a possible explanation for the different responses of stressed tomato to PGPR-T3 at the early growth stage and at crop termination is that the stress conditions might have decreased the population density of PGPR in the long term. In agreement with this consideration, Patakioutas et al. (2015) reported an appreciable reduction in the population density of *Bacillus amyloliquefaciens* in a hydroponic greenhouse tomato crop after about 30 weeks of cultivation. This finding indicates that inoculation with some PGPR strains may need repetition one or more times in long-term greenhouse crops exposed to stress conditions to maintain an effective density of PGPR population in the root zone. However, this has to be confirmed experimentally. On the other hand, PGPR-T1 had no protective effect against combined nutrient/water stress during the early vegetative period of growth but maintained shoot growth to similar levels with non-inoculated non-stressed plants at crop termination. This response indicates that the mix of the *Enterobacter* sp. strains C1.2 and C1.5 deploys a different protective mechanism against stress than the rest of the PGPR treatments tested in the current study, which is adaptive and operates only under long-term stress conditions.

Grafting onto ‘M82’ did not protect plants against combined nutrient and water stress, in agreement with Rigano et al. (2016), who found that this rootstock is not tolerant to water deficit, although it is a vigorous rootstock. However, under non-stress conditions, grafting onto ‘M82’ boosted shoot growth, in agreement with Ntatsi et al. (2014) and Rouphael et al. (2017), who stated that grafting tomato onto vigorous rootstocks increases tomato growth.

### Tomato Yield

Several studies have shown that PGPR can increase tomato fruit production, an effect that in most cases is ascribed to enhanced protection against plant diseases. For instance, Gravel et al. (2007) reported yield increases in hydroponic tomato inoculated with *Pseudomonas brevicompactum*, *Pseudomonas marginalis*, *Pseudomonas putida*, and *Trichoderma atroviride*. In other studies, PGPR enhanced tomato fruit yield only under stress conditions. Thus, Gagné et al. (1993) reported yield increases in an autumn crop of tomato inoculated with a *Pseudomonas* fluorescence strain under suboptimal natural light conditions. However, in spring, under more favorable light conditions, the tomato fruit biomass was not influenced by inoculation with *Pseudomonas*. In the current study, combined water and nutrient stress decreased significantly tomato fruit biomass, whereas PGPR inoculation under the conditions of the current experiment was incapable of eliminating or mitigating the stress effects on fruit production. The lack of any benefit from PGPR application concerning fruit yield supports the previously stated notion that the population density of PGPR in the roots decays with time under real growing conditions, and re-inoculation during a long-term cropping period is required. This suggestion is in line with a previous report of Yan et al. (2003), who found that the population density of *Pseudomonas* strains used as PGPR in the roots followed a decreasing pattern with time.

Grafting onto M82 increased total fruit yield, regardless of stress conditions, whereas the Extra Class and Class I fruit yield was increased by grafting only under non-stress conditions. These results show that the fruit biomass production in tomato grafted onto ‘M82’ is less susceptible to combined nutrient/water stress than the vegetative growth. However, these results also corroborate previous results of Rigano et al. (2016), suggesting that ‘M82’ is not tolerant to water stress.

### Leaf Nutrient Status

The reduction of the total N concentration in leaves of tomato exposed to combined nutrient and water stress is fully anticipated since one of the stress factors was the reduction of the N supply by 50%. However, under combined stress conditions, PGPR-T2, PGPR-T3, and PGPR-T4 managed to sustain the leaf N concentrations to similar levels with those measured in non-stressed plants. These results show that the PGPR strains DN1.2 of *Paenibacillus* sp., C3.1 of *E. mori*, and D2.4 of *Lelliottia* sp. are capable of maintaining normal nitrogen uptake under conditions of restricted N supply up to 50% of the standard levels. In a previous study, Adesemoye et al. (2010) found that enhanced N uptake by plants was due to increased plant uptake of N originating from fertilizer and not from enhanced mobilization of organic N reserves in the soil. The current results are in line with those findings, since in the current study the plants were cultivated on an inert medium in which only inorganic N was supplied. Cordero et al. (2018) also reported increased N concentration in tomato leaves following root inoculation with PGPR. According to these researchers, increased N acquisition by plants treated with PGPR results from increased root surface, stimulation of metabolic processes contributing to nutrient mobilization, and utilization of N reserves from the microbial biomass turnover. Nevertheless, the maintenance of normal leaf N in tomato plants inoculated with PGPR-T2, PGPR-T3, and PGPR-T4 under combined stress conditions did not prevent stress-induced fruit yield restrictions in these treatments. These results indicate that the yield reduction in tomato exposed to combined nutrient and water stress was not due to shortage of N.

Unlike the leaf N, the leaf P concentrations were not affected by PGPR, presumably because, in the current study, tomato was cultivated hydroponically on an inert medium and not in the soil. The P concentrations in NSs supplied to hydroponic crops (usually 1–1.5 mmol L^–1^, Savvas and Gruda, 2018) may be 100-fold higher than the Pi concentrations encountered by plants in the soil solution (Schachtman et al., 1998), while the inert porous media used in hydroponics do not contain any P reserves. Thus, PGPR could not provide a benefit by mobilizing P reserves under conditions of limited P availability in the root environment. In agreement with this consideration, Gravel et al. (2007) reported that inoculation of tomato roots with PGPR had no impact on the leaf nutrient status in tomato plants grown on rockwool, whereas the same microorganisms increased the available P in plants cultivated on an organic medium. Furthermore, the same researchers did not find any beneficial effect of PGPR on plant K status, in agreement with the current study.

### Metabolomics Analysis

The metabolomics analysis in tomato leaves revealed a clear discrimination between the different treatments, which indicates that they all had an impact on leaf metabolism. PGPR-T3 exerted the strongest impact on leaf metabolism, as all PGPR-T3 treatments, irrespective of grafting and stress exposure, were clearly discriminated from the treatments without PGPR inoculation (Figures 2, 3). The only exception was the treatment combining PGPR-T3 inoculation with grafting onto ‘M82’ under water/nutrient stress, which exhibited a similar metabolic profile with that of the non-inoculated treatments, thus questioning the successful symbiosis of PGPR-T3 with the roots of M82.

Plants are complex organisms, and it is difficult to study and understand the exact role of each of the identified metabolites. In general, the metabolic roles of most sugars, proline, α-α-trehalose, monopalmitin, and GABA have been extensively studied. Other metabolites, for example, those participating in the TCA cycle, have also been studied extensively, but the exact function of most of them is poorly understood (Semel et al., 2007). Although many studies corroborate previous findings concerning metabolic pathways, the role of many metabolites remains contentious among researchers (Van Der Merwe et al., 2009).

The metabolic responses of plants differ when they are exposed only to water, N, or P shortage, compared with cultivation under stress caused by combined shortage. In the present study, glycine, for example, increased in plants exposed to combined water, N, and P shortage. Sung et al. (2015), who studied the impact of N, P, and K deficiencies on leaf and root metabolism in tomato, found that glycine decreases in tomato leaves exposed to partial N starvation but increases in plants exposed to P deficiency. Combined consideration of the current results and those of Sung et al. (2015) leads to the conclusion that, in the current study, the impact of P stress on glycine overwhelmed that of N stress. In contrast, citrate decreased in the present study under combined N and P stress, similarly to what Sung et al. (2015) reported under N stress, but diverged from the results under P shortage, indicating that the dominant factor for citrate in the current study was the N availability. Glycerate and fructose were reduced by combined stress in the present study, similarly to the reduction found by the same authors under only N or P stress. The observed accumulation of malate and trehalose under combined stress could be ascribed to their well-documented protective role under osmotic and oxidative stress (Cortina and Culiáñez-Macià, 2005; Semel et al., 2007). Finally, myo-inositol can be a protective compound under salt stress conditions, with a dual role. On the one hand, it can affect metabolism as a signal or a key metabolite (Hu et al., 2018). On the other hand, according to previous studies, under salinity stress, myo-inositol can balance osmotically the cytosol (Zhang et al., 2001), while its derivatives participate in plant stress responses such as programmed cell death (Kaur et al., 2013). However, in the present study, the levels of myo-inositol decreased in plants exposed to combined water, N, and P stress compared with non-stress conditions. This response may indicate that myo-inositol has a protective role only against salt stress, while the genes regulating its biosynthesis are downregulated under nutrient or water shortage conditions.

The phosphoric acid determined as a metabolite represents all forms of phosphates participating in cell metabolism. As reported by Wasaki et al. (2003), P deficiency imposes upregulation of many genes related to P metabolism and concomitantly enhances the biosynthesis of metabolites involved in P metabolism, such as pyruvate kinase and the accompanying phosphoenolpyruvate phosphatase, glucose-6-phosphate isomerase, inorganic pyrophosphatase, purple acid phosphatase, and inorganic phosphate transporters. Nevertheless, P deficiency also downregulates genes related to P metabolism, such as acid phosphatase (Wasaki et al., 2003). Under stress conditions, the needs for energy transport aiming to mobilize defense mechanisms entail increased biosynthesis of phosphorylated intermediates (Latz et al., 2013). As a result, upregulation of genes involved in the biosynthesis of phosphate containing metabolites exceeds downregulation, and concomitantly, the level of metabolically related phosphates increases, although the total P concentration in plant tissues may decrease, as was the case in the current study (Table 2). In agreement with this consideration, Wasaki et al. (2003) stated that P deficiency stress stimulates upregulation of genes that control the biosynthesis of metabolites substituting phospholipids in cell membrane, thereby increasing the levels of phosphates available for metabolic functions that contribute to energy transport. Phospholipids count for 17% of P in leaves (Lambers et al., 2012). Thus, their substitution with non-phosphorus lipids in cell membranes in response to stress conditions (Tawaraya et al., 2018) could maintain the levels of P-containing metabolites.

Colonization of tomato roots by PGPR clearly affected tomato metabolism, but only few metabolites exhibited a substantial fluctuation. The strongest impact of PGPR was on trehalose biosynthesis. Trehalose, which serves primarily as a storehouse of glucose for energy and/or for synthesis of cellular components, is also involved in functions protecting membranes and proteins against stress (Elbein et al., 2003). Furthermore, trehalose is involved in interactions between plants and microorganisms, including both symbiosis (Müller et al., 1994) and pathogenesis (Brodmann et al., 2002). More specifically, trehalose is present at relatively high concentrations in nodules, indicating a positive relationship between trehalose and nodulation (Streeter and Gomez, 2006), while the suppression of trehalose metabolism is associated with reduced pathogenicity (Tournu et al., 2013). These results indicate that trehalose promotes colonization of plants by bacterial microorganisms through alteration of the carbohydrate metabolism to their favor, regardless of whether the plant–microbe relationship is beneficial or destructive to plants. Hence, the accumulation of trehalose in plants treated with PGPR-T3 in the current study, compared with non-treated plants, is presumably part of a mechanism contributing to successful establishment of a plant/PGPR symbiosis.

Rudrappa et al. (2008) have reported that malic acid/malate is a metabolite-promoting plant–PGPR communication in roots. Rudrappa et al. (2008) performed experiments with knockout *Arabidopsis* mutants for malic acid transporter and observed that the plants were unable to recruit PGPR for symbiosis. The recorded downregulation of malate in leaves of tomato inoculated with PGPR may be a result of malate translocation from leaves to roots aiming to facilitate colonization by PGPR. However, the metabolic profile of roots was not determined in the current study, and thus this hypothesis, although reasonable, is not adequately supported by the current experimental data.

Monopalmitin is a carbon source for plants, plant-related microbiome, and legume plants to sustain symbiosis with arbuscular mycorrhizal fungi, which is found in root exudates of several plant species (Feng et al., 2019). In sugarcane leaves, 2-monopalmitin content increased when inoculation with PGPR (*Gluconacetobacter diazotrophicus* and *Herbaspirillum seropedicae*) was combined with humic acid addition, whereas PGPR inoculation itself had no effect on monopalmitin accumulation (Aguiar et al., 2018). However, little is known on the exact role of monopalmitin, and although it was reduced in the leaves of plants colonized by PGPR, this metabolite in roots might be involved in plant/PGPR symbiosis.

Grafting onto the rootstock ‘M82’ had either a very weak or no impact on most metabolites. However, the levels of three metabolites were clearly reduced by grafting onto ‘M82’ (malate, D-fructose, and myo-inositol), while one metabolite (oleic acid) accumulated. According to Van Der Merwe et al. (2009), transgenic tomato exhibiting decreased expression of the mitochondrial malate dehydrogenase, and concomitantly increased malate levels, imposed a clear reduction in root biomass. In the current study, the increased root biomass of grafted plants was associated with reduced malate levels, corroborating an inverse relationship between malate levels and root biomass. The reduction of the D-fructose levels in leaves of tomato grafted onto ‘M82’ compared with self-grafted plants may point to an increased sink activity in roots, as many tomato rootstocks form a more vigorous root system than self-rooted plants (Rouphael et al., 2017). Oleic acid is one of the three fatty acids (along with palmitic and linoleic acid) present, as free fatty acids in tomato fruits, melons, and cucumbers and are strongly responsible for the flavor of many vegetables, as they act as precursors in the biosynthesis of most straight-chain esters in aromatic volatile compounds (Hao et al., 2018). Having a profile different than that of fruits, tomato leaf fatty acids are responsible for leaf flavor, producing aroma volatiles through the lipoxygenase/hydroperoxide lyase enzyme pathway. Thus, changes in fatty acid composition in tomato leaves can lead to altered composition of lipid-derived flavor compounds (Wang et al., 2001). The results obtained from the current study do not reveal how the genotypic difference between root and leaves in grafted plants mediates a change in the level of oleic acid in tomato leaves. Furthermore, the current study does not show if the levels of fatty acids associated with flavor were influenced also in fruit of grafted tomato. However, Hao et al. (2018) reported that the levels of free fatty acids in fruit of grafted melons were lower than in fruits from non-grafted plants, and this was associated with reduced fruit aroma. Further research is required to test the fatty acid profile in fruit of grafted tomato and relate it to possible changes in volatile compounds and concomitantly in fruit flavor.

## Conclusion

The improved vegetative growth of plants inoculated with all PGPR strains tested in this study, but especially with *E. mori* strain C3.1, corroborates previous studies, which showed that application of appropriate PGPR strains may improve tomato growth. On the other hand, the lack of any beneficial effect of PGPR on fruit yield, but also on the vegetative growth at the end of the crop under combined stress conditions, indicate that a single PGPR inoculation at crop establishment may be not sufficient, especially under stress conditions. Selection of appropriate PGPR strains, tolerant to multiple stress, and repetition of PGPR application during the cropping period, are key factors to obtain benefits from PGPR application, especially under stress conditions.

The improved growth and yield of non-stressed tomato plants grafted onto ‘M82,’ compared with non-grafted plants and the absence of any yield benefit from grafting onto this rootstock under stress conditions, corroborate previous reports suggesting that grafting onto suitable rootstocks enhances tomato fruit yield. However, on the other hand, the results of the current study show that ‘M82’ is not a suitable rootstock for plants grown under conditions of restricted water supply and points to the need to screen for tolerant rootstock genotypes to water and nutrient limitations.

The metabolomics analysis showed that water/nutrient stress and PGPR inoculation have a strong impact of the leaf metabolism, whereas grafting has a weaker but non-negligible impact. Combined nutrient and water stress enhanced by 10% or more the biosynthesis of L-lactic acid, ethyl phosphoric acid, phosphoric acid, glycine, malate, oleic acid, and α-α-trehalose, while it reduced by 10% or more the levels of succinic acid, glyceric acid, citric acid, D-fructose, myo-inositol, stearic acid, and 1-monopalmitin. Similarly, the application of PGPR enhanced by 10% or more D-fructose and α-α-trehalose, while it reduced by 10% or more the biosynthesis of ethanolamine, glycerol, L-serine, malate, myo-inositol, and 1-monopalmitin. Finally, grafting onto M82 enhanced by 10% or more the biosynthesis of oleic acid, while reducing by 10% or more the biosynthesis of malate, D-fructose, and myo-inositol compared with self-grafting.

As a general conclusion, this study showed that both PGPR and grafting are promising tools for improvement of tomato performance. However, to obtain a benefit under combined stress conditions, selection of appropriate PGPR strains and rootstock genotypes, and establishment of suitable protocols regarding method, time, and number of PGPR applications are crucial factors for success. The current study contributes to a better understanding of changes imposed to plant metabolism by combined water/nutrient stress, PGPR, and grafting. This new insight concerning metabolites influenced by stress, PGPR, and grafting can be useful in rapid screening of PGPR strains and rootstock genotypes with resilience to multiple stress conditions, which may increasingly appear in commercial tomato crops in view of the climate change.

## Data Availability Statement

The raw data supporting the conclusions of this article will be made available by the authors, without undue reservation.

## Author Contributions

DS and PK conceived and designed the experiments. PK, GN, GM, ES, and IK performed the experiments and the mineral analyses. KA and PK performed the metabolomics analysis. AT provided the PGPR strains and performed the inoculation. PK, DS, and KA analyzed the data and wrote the manuscript. DS, KA, GN, AT, and IK reviewed the manuscript. All authors have read and approved the manuscript.

## Conflict of Interest

The authors declare that the research was conducted in the absence of any commercial or financial relationships that could be construed as a potential conflict of interest.

## Footnotes

1 https://www.aua.gr/pesticide-metabolomicsgroup/Resources/default.html

## Supplementary Material

Supplementary Figure 1 Orthogonal partial least squares-discriminant analysis (OPLS-DA) PC1/PC2 score plot of metabolomic profiles of non-stressed and exposed to combined stress tomato plants **(i)**, non-inoculated plants (No PGPR) and plants inoculated with PGPR-T3 **(ii)**, and self-grafted tomato plants (‘Belladonna’ F1) and plants grafted onto ‘M82’ **(iii)**. The ellipse represents the Hotelling T2 with 95% confidence interval.

Supplementary Data set 1 Annotated metabolite features in the GC/EI/MS profiles of *Solanum lycopersicum* L. leaves.
