# Supplementary figures and images for: Impact of Plant Growth-Promoting Rhizobacteria Inoculation and Grafting on Tolerance of Tomato to Combined Water and Nutrient Stress Assessed via Metabolomics Analysis
Source: Front Plant Sci. 2021 Jun 4;12:670236. doi: 10.3389/fpls.2021.670236 (PMC8212936; doi:10.3389/fpls.2021.670236)

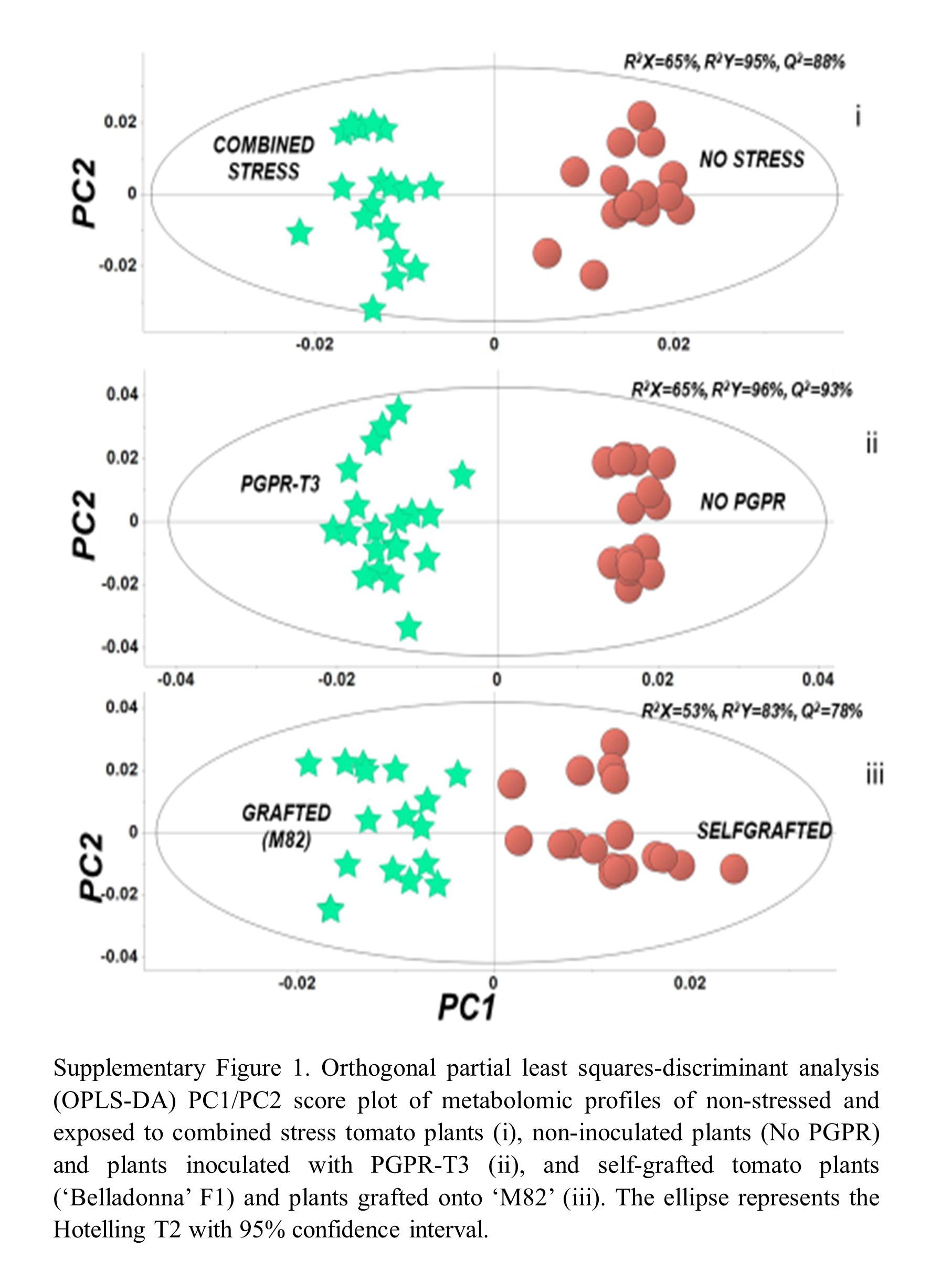

Supplement: Supplementary Figure 1 — Orthogonal partial least squares-discriminant analysis (OPLS-DA) PC1/PC2 score plot of metabolomic profiles of non-stressed and exposed to combined stress tomato plants (i), non-inoculated plants (No PGPR) and plants inoculated with PGPR-T3 (ii), and self-grafted tomato plants (‘Belladonna’ F1) and plants grafted onto ‘M82’ (iii). The ellipse represents the Hotelling T2 with 95% confidence interval. [file Image_1.TIF]
