# Supplementary material for: Impact of Plant Growth-Promoting Rhizobacteria Inoculation and Grafting on Tolerance of Tomato to Combined Water and Nutrient Stress Assessed via Metabolomics Analysis
Source: Front Plant Sci. 2021 Jun 4;12:670236. doi: 10.3389/fpls.2021.670236 (PMC8212936; doi:10.3389/fpls.2021.670236)
Supplement: Supplementary file 3 [file Table_1.docx]

**Supplementary Table 1.** Nutrient concentrations in the nutrient solution supplied to non-stressed plants and plants exposed to combined water and nutrient stress (50% reduction of water, N and P supply) during the vegetative and reproductive growth phase.

| Nutrient | No stress | |  | Combined stress | | Unit |
| --- | --- | --- | --- | --- | --- | --- |
|  | Vegetative phase | Reproductive phase |  | Vegetative phase | Reproductive phase |  |
| NO_3_^-^ | 14.00 | 12.50 |  | 7.00 | 6.25 | mM |
| K^+^ | 7.00 | 8.00 |  | 7.00 | 8.00 | mM |
| Ca^2+^ | 5.10 | 5.00 |  | 5.10 | 5.00 | mM |
| Mg^2+^ | 2.40 | 2.50 |  | 2.40 | 2.50 | mM |
| SO_4_^2-^ | 5.40 | 5.40 |  | 5.40 | 5.40 | mM |
| H_2_PO_4_^-^ | 1.50 | 1.50 |  | 0.75 | 0.75 | mM |
| NH_4_^+^ | 1.50 | 1.50 |  | 0.75 | 0.75 | mM |
| Fe | 15.00 | 15.00 |  | 15.00 | 15.00 | μM |
| Mn | 10.00 | 10.00 |  | 10.00 | 10.00 | μM |
| Zn | 5.00 | 5.00 |  | 5.00 | 5.00 | μM |
| B | 30.00 | 30.00 |  | 30.00 | 30.00 | μM |
| Cu | 0.80 | 0.80 |  | 0.80 | 0.80 | μM |
| Mo | 0.50 | 0.50 |  | 0.50 | 0.50 | μΜ |
